# Identifying Transcripts with Tandem Duplications from RNA-Sequencing Data to Predict BRCA1-Type Primary Breast Cancer

**DOI:** 10.3390/cancers14030753

**Published:** 2022-01-31

**Authors:** Shuoying Qu, John W. M. Martens, Antoinette Hollestelle, Marcel Smid

**Affiliations:** Department of Medical Oncology, Erasmus MC Cancer Institute, University Medical Center Rotterdam, 3015 GD Rotterdam, The Netherlands; s.qu@erasmusmc.nl (S.Q.); a.hollestelle@erasmusmc.nl (A.H.); m.smid@erasmusmc.nl (M.S.)

**Keywords:** BRCA1-type breast cancer, homologous recombination repair deficiency, tandem duplication, RNA sequencing, classification algorithm, cancer driver enrichment, *PTEN* inactivation

## Abstract

**Simple Summary:**

Homologous recombination repair deficiency (HRD) is a biomarker for the response to PARP inhibitor anti-cancer treatment. Therefore, methods that detect the HRD phenotype in cancers in a (cost-)effective manner are pivotal. In this respect, the HRDetect and CHORD algorithms were developed to classify (the type of) HRD cancers from whole genome sequencing data. In addition, functional assays have also been established, but these require fresh cancer tissue. Here we present a novel method to specifically classify BRCA1-type HRD from RNA-sequencing data with high sensitivity. BRCA1-type cancers typically display small (<10 kb) tandem duplications, in contrast to BRCA2-type cancers. By detecting these small TDs among transcripts, we increase the toolbox for detecting HRD with a method that does not require whole genome sequencing of both tumor and normal tissue.

**Abstract:**

Patients with cancers that are deficient for homologous recombination repair (HRD) may benefit from PARP inhibitor treatment. Therefore, methods that identify such cancers are crucial. Using whole genome sequencing data, specific genomic scars derived from somatic mutations and genomic rearrangements can identify HRD tumors, with only BRCA1-like HRD cancers profoundly displaying small (<10 kb) tandem duplications (TDs). In this manuscript we describe a method of detecting BRCA1-type HRD in breast cancer (BC) solely from RNA sequencing data by identifying TDs surfacing in transcribed genes. We find that the number of identified TDs (TD-score) is significantly higher in BRCA1-type vs. BRCA2-type BCs, or vs. HR-proficient BCs (*p* = 2.4 × 10^−6^ and *p* = 2.7 × 10^−12^, respectively). A TD-score ≥2 shows an 88.2% sensitivity (30 out of 34) to detect a BRCA1-type BC, with a specificity of 64.7% (143 out of 221). Pathway enrichment analyses showed genes implicated in cancer to be affected by TDs of which *PTEN* was found significantly more frequently affected by a TD in BRCA1-type BC. In conclusion, we here describe a novel method to identify TDs in transcripts and classify BRCA1-type BCs with high sensitivity.

## 1. Introduction

The incidence of breast cancer (BC) is regarded as the highest among malignancies in women worldwide and is still climbing by 0.3% per year [1]. One of the strongest risk factors for developing BC is a family history of the disease [2]. About 20–25% of familial BC patients inherit mutations in BC susceptibility genes *BRCA1*, *BRCA2*, *PALB2*, *CHEK2* or *ATM*. Germline mutations in *BRCA1* are associated with a cumulative BC risk to age 80 of 72% (95% Confidence Interval (CI) = 65–79%) [3]. Therefore, *BRCA1* is regarded as a high-risk BC susceptibility gene [4]. 

The protein encoded by *BRCA1* has three different domains. The first is the RING domain, which is positioned on the amino-terminal end of the protein and functions as an E3 ubiquitin ligase. The second is the BRCT domain, of which two are located on the carboxy-terminal end of the protein and function as phosphopeptide recognition modules [5]. The third is the serine containing domain (SCD), which contains a large number of phosphorylation sites and gets phosphorylated by ATM/ATR kinases upon DNA damage [6]. With these different domains, BRCA1 is equipped with multiple functions, consisting of interacting with other tumor suppressors, cell cycle checkpoint regulation and mediating DNA repair [4]. 

Genomic instability is a core factor driving tumor development. In this respect, homologous recombination (HR) repair is one of the main DNA double-strand break (DSB) repair pathways together with the non-homologous end joining (NHEJ) pathway [7]. HR plays a prominent role in maintaining genome integrity by providing high-fidelity repair using a copy of the intact sister chromatid during the S and G2 phases of the cell cycle in proliferating cells [4]. Since BRCA1 and BRCA2 proteins serve a purpose of maintaining genome integrity through HR-mediated DNA repair, mutations disrupting BRCA1 or BRCA2 function result in a HR-deficiency (HRD) phenotype [8]. As a consequence, DSBs are repaired by the error-prone NHEJ pathway, resulting in accumulation of genetic alterations [9]. Since HRD and inhibition of PARP are synthetically lethal, cancer patients carrying *BRCA1* or *BRCA2* mutations benefit from PARP inhibitor therapy [10]. However, not all HRD-associated BCs have a genetic defect in either *BRCA1* or *BRCA2* [11], therefore identifying the HRD phenotype itself among cancers is crucial [9].

Recent developments in whole genome sequencing (WGS) have made it possible to recognize HRD in BC by identifying specific genomic ‘scars’: signatures based on single base substitutions (SBSs), small insertions and deletions (IDs) or rearrangements [12,13]. Previous studies have shown that SBS signature 3 and 8 as well as ID signatures 6 and 8 are associated with HRD [8,12,13]. Moreover, BRCA2-deficient tumors are associated with rearrangement signature 5, whereas BRCA1-deficient tumors are associated with rearrangement signature 3, enabling subclassification among HRD tumors. The BRCA1-associated rearrangement signature 3 is mainly characterized by small tandem duplications (TDs, <10 kb), a property thus not identified among BRCA2-deficient BC [14]. Loss of BRCA1 function allows for these TDs to arise at stalled replication forks through a replication restart-bypass mechanism. This mechanism is terminated by either end joining or microhomology-mediated template switching, the latter resulting in complex TD breakpoints [15].

To identify HRD using WGS data, two algorithms were developed. First HRDetect [8] was described which uses 6 genomic parameters, among which rearrangement signatures 3 and 5. This algorithm is designed to predict the HRD status (i.e., deficient or proficient), not to distinguish between BRCA1- and BRCA2-deficient HRD. A second model named Classifier of Homologous Recombination Deficiency (CHORD) was also described to identify HRD status. CHORD uses 29 genomic features, grouped into SBS, ID and rearrangement signatures and is additionally able to distinguish BRCA1- from BRCA2-type HRD [16].

Here we aimed to use RNA sequencing (RNAseq) data as a source to identify genomic TDs, the characteristic structural variant (SV) that is strongly associated with BRCA1-deficient BC [14]. We describe a method to detect transcripts carrying TDs in RNAseq data, which we compared with HRDetect and CHORD to evaluate how well RNAseq based TDs mimic HRD predictions by these algorithms. Furthermore, we identify which genes are affected by a TD of which *PTEN* is the most significant recurrent target.

## 2. Materials and Methods

### 2.1. Sequencing Data

RNAseq data were generated at our lab for the BASIS consortium [14,17] for a subset of 266 primary BCs from a total of 560 that were whole genome sequenced. Genome and transcriptome data are available through the European Genome Phenome Archive under accession number EGAS00001001178. Internal Review Boards of each participating institution approved collection and use of samples of all patients in this study. Sequence protocols of the samples were previously described in detail [14]. In short, total RNA after genomic DNA removal, clean-up and depletion of ribosomal RNA using Duplex Specific Nuclease treatment, was used as input for random-primed cDNA synthesis. Library preparation and sequencing was performed as described [14] and paired-end (75 bases) sequencing was performed on an Illumina HiSeq 2000. The resulting fastq files were mapped to the human reference genome GRCh38 using STAR version 2.4.2a [18] and the resulting bam files were sorted and indexed using Sambamba version 0.6.6 [19]. Gene annotation was derived from GENCODE Release 23 (https://www.gencodegenes.org/ accessed on 16 June 2021).

### 2.2. Identification of TDs

**Figure 1:**
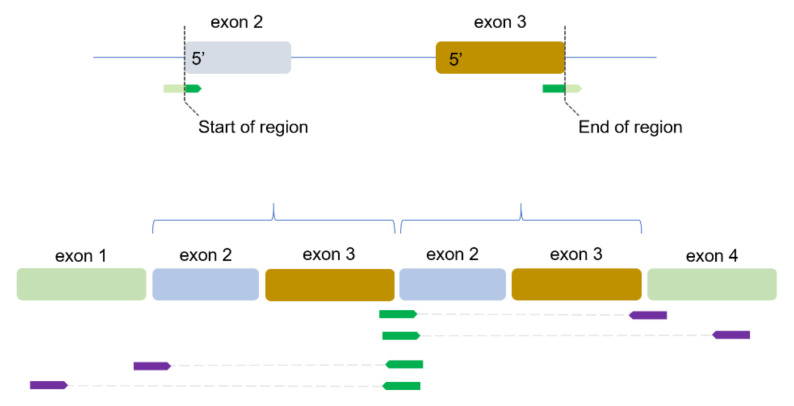
Identifying transcripts with a TD region. Top panel schematically shows how a junction read (green) would be located on the DNA reference. Introns are shown as horizontal lines. The 5′ of the junction read maps to the 3′ end of exon 3, while the remainder of the read maps to the 5′ of exon 2. Since this is an unexpected orientation and depending on where the majority of the read maps, the read is annotated as mapping to one of the boundaries. The unmapped bases are annotated as soft clipped (S in the CIGAR score). The chromosomal positions of the junction ‘breakpoints’ are used as the start and end of a region. The bottom panel shows a TD of exon 2–3, with the read mates (purple) are partly or completely located outside the region defined by the chromosomal position of the junction read locations.

To find sequence reads that identify TDs, a two-stage process is used. First, the locations where so-called junction reads are mapped and used as candidate TD regions (see Figure 1). In RNAseq, reads can span multiple exons and when sequencing total RNA reads may also map to intron sequences. Junction reads are defined here as those that map to a non-canonical gene structure. This would be a read where the 5′ end is mapped to for example the 3′ end of exon 3, with the remaining bases in the read mapping to exon 2 (where exon 4 or intron 3 would be expected, see top panel Figure 1). Such a configuration would usually indicate a circular molecule, and the genes that produce these circular RNAs (circRNAs) were identified in our previous paper [20]. Of note, it is not required that multiple exons are involved, this also applies to a single exon. However, junction reads can also be derived from genes that harbor TDs, therefore, in the second stage, the read-mate of the junction read is evaluated. If a read-mate maps within the region bounded by the 5′ and 3′ mapping locations of the junction read it could be either a circRNA or a TD configuration [20]. However, if the read-mate of a junction read maps outside the region then this read-pair is considered a TD read-pair (bottom panel Figure 1). For each sample, the number of regions that show evidence of TD read-pairs is used as the TD-score.

In detail, a Perl script was developed to evaluate which of the junction regions identified by our method to identify circRNAs have read-pairs indicative of a TD. First, for each sample only those junction regions exactly mapping to exon boundaries of the same gene were included. For these selected regions, the reference sequence GRCh38 is used to construct a virtual junction sequence by taking the first and last 100 nucleotides of the region and concatenating these last to first. Next, RNAseq sequence reads that are mapped to the region are selected when uniquely mapped and of high mapping quality (MAPQ = 255 in STAR notation). When more than 4000 reads are present in the region, only the first and last 2000 reads (mapping to the 5′ and 3′ end of the region) are included. The sequence of these reads is re-mapped to the virtual junction sequence. This is done since the desired junction reads are mapped by STAR but are soft clipped. For example, a 75-base read can uniquely map with bases 1–50 to the 3′ end of a region with the last 25 bases annotated as soft clipped (Figure 1). This information is contained within the Concise Idiosyncratic Gapped Alignment Report (CIGAR) score and would be annotated as 50M25S. If these 25 bases exactly match the 5′ end of the region then the read is fully complimentary to the virtual junction sequence. A minimum of 10 clipped bases is required to prevent a match to the junction sequence by chance. Since the majority of the read is already uniquely mapped to that region, 10 bases fully matching to the rest of the junction sequence is considered sufficient. Next, the read mate of this junction read is evaluated if it maps outside the region (indicated in top panel of Figure 1) using the insert size (ISIZE) and position of read mate (MPOS) fields. If so, then the read pair is considered indicative for a TD. Four scenarios are considered (Figure 1): a forward read (SAM flag 163 or 99) mapped to the 5′ or 3′ end of a region with a reverse read mate mapped further than the 3′ end of the region, and conversely, a reverse read (flag 83 or 147) mapped to the 5′ or 3′ end of a region with a forward read mate mapped before the 5′ start of a region.

### 2.3. Enrichment Analysis

To determine if genes affected by a TD have functional roles in common, an over-representation analysis was performed using DAVID [21]. Gene Ontology, KEGG and Biocarta databases were used for functional annotation. Pathways were considered significant when the false discovery rate (FDR) corrected *p*-value was below 0.05.

### 2.4. Statistical Analyses

STATA v14 (StataCorp, College Station, TX, USA) and R v4.0.3 (https://www.R-project.org/ accessed on 27 November 2020) were used to perform the statistical tests that are indicated in the text. *p*-values are two sided and corrected for multiple testing using the Hochberg method where necessary and were considered significant below 0.05.

## 3. Results

Small TDs of mainly 1–10 kb in size are one of the genomic scars that BRCA1-type BCs uniquely produce [14]. In this study, we investigated whether these small TDs can be identified in the transcriptome, using RNAseq data from a total of 266 primary BCs with matching WGS data available. For these samples the HRD status was also available as determined by HRDetect [8] and the CHORD algorithm [16]. Both HRDetect and the CHORD algorithm are capable of distinguishing HRD from HR-proficient (HRP) samples and classified 60 samples as HRD by HRDetect and 51 samples as HRD via CHORD with 50 samples overlapping. HRDetect called 195 samples as HRP, while CHORD called 204 samples as HRP with 194 samples overlapping. In addition, CHORD is also able to differentiate between BRCA1- and BRCA2-type HRD. 

We aimed to identify TD regions using RNAseq data by finding sequence reads that map to non-canonical exon-exon junctions that arises when an intragenic part is duplicated consecutively (Figure 1). For each sample, the number of identified TD regions is used as a TD-score. We used the TD-score as a classifier to identify the BRCA1-type HRD phenotype as established by CHORD. Furthermore, we also studied the gene transcripts that were (recurrently) affected by a TD in samples with a BRCA1-type phenotype.

### 3.1. Abundance of TD Regions

**Figure 2:**
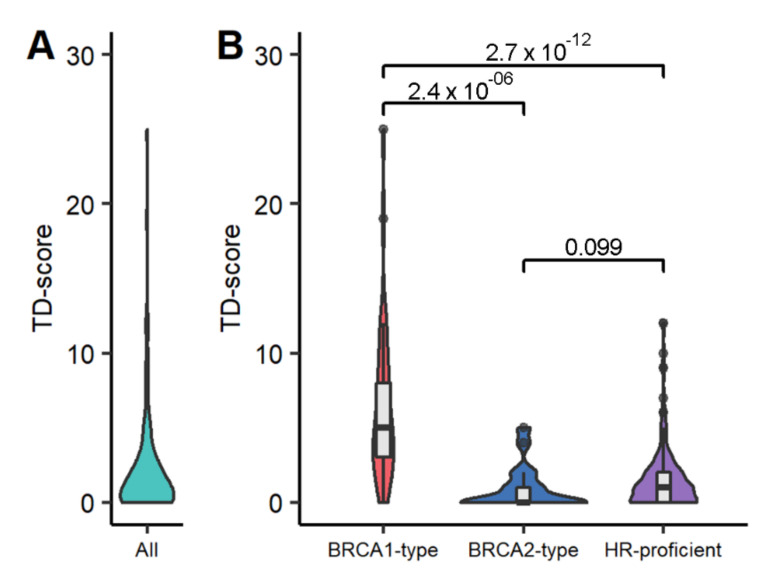
Distribution of TD-score in all samples (**A**) and by CHORD-call (**B**). *p*-values are from Mann–Whitney U-test. Sample sizes are *n* = 34, *n* = 17 and *n* = 204 for BRCA1- and BRCA2-type HRD and HRP, respectively.

In total, 565 TD regions were identified in 266 primary BC samples (Table S1). In almost a third of the samples (*n* = 84, 31.6%) we did not observe any TDs in the RNAseq data. Overall, the per-sample average is 2.12 (95% CI = 1.75–2.50) and the amount of TDs per sample ranges from 0–25 (Figure 2A). We evaluated if these TDs found in RNA were also reported in the DNA of the same sample: 64% (105 out of 164) of the TDs found in RNAseq data matched with a reported TD in DNA (Table S1). Next, we associated the TD-score with CHORD classification. According to CHORD, 34 samples are BRCA1-type (13%), 17 are BRCA2-type (6%) and 204 samples are HRP (77%) with the remaining 11 samples without CHORD-call (‘cannot be determined’). Figure 2B clearly shows that the TD-score is significantly higher in the BRCA1-type samples (Mann–Whitney U-test [MWU] *p* = 2.4 × 10^−6^ and *p* = 2.7 × 10^−12^ for BRCA1-type vs. BRCA2-type or HRP, respectively). The median TD scores are 5, 0 and 1 for BRCA1-type, BRCA2-type and HRP BCs, respectively. The TD score was also significantly higher in the HRD compared to HRP BCs based on the HRDetect calls (MWU *p* = 8.9 × 10^−10^, Figure S1A) and the *BRCA1* mutant BCs versus the *BRCA2* mutant or wild-type BCs (MWU *p* = 8.3 × 10^−3^ and *p* = 2.0 × 10^−4^ respectively, Figure S1B). Finally, we also verified the distribution of the TD-score among rearrangement signature-derived clustering of primary BC reported by Nik-Zainal et al. in their Figure 5 [14]. Cluster D, which consists of almost all *BRCA1*-mutated samples and having a profound number of TDs at the genomic level, showed a significantly higher TD-score compared to all other groups (MWU all *p* < 1.6 × 10^−4^, Figure S1C).

### 3.2. Power of the TD-Score to Predict BRCA1-Type HRD 

**Figure 3:**
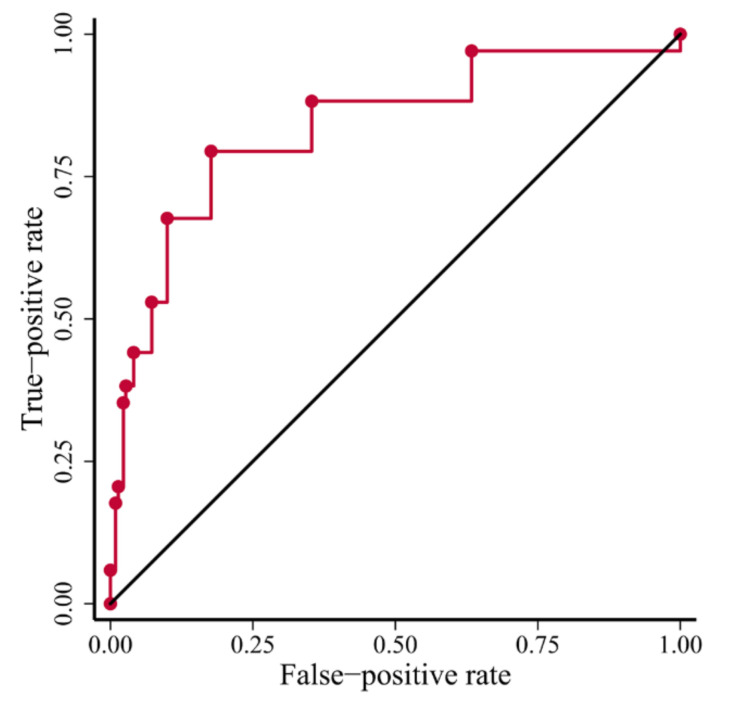
ROC curve of TD score to predict a BRCA1-type BC. Diagonal black line indicates a diagnostic test with performance no better than chance.

To evaluate how well the TD score, based on RNAseq, approximates the BRCA1-type call as assigned by CHORD using WGS data, we performed a receiver operating characteristic (ROC) analysis (Figure 3). We labelled the BRCA1-type BCs as one group, with the other group containing both the BRCA2-type and HRP BCs. The area under the curve (AUC) for the TD score is 0.87 (95% CI = 0.80–0.93, *p* = 1 × 10^−27^), thus showing a high predictive power to classify these groups. When using a cut-off of ≥2 TD-regions, the sensitivity to detect a BRCA1-type BC is 88.2% (30 out of 34) with a specificity of 64.7% (143 out of 221). Figure S1D shows an overview of the TD score and *BRCA1/2* bi-allelic inactivation status in CHORD-positive cases. Since the majority of *BRCA1*-mutated patients are diagnosed with ER-negative BC, another use-case for the TD score would be in this specific subgroup [22]. Therefore, within ER-negative samples (*n* = 74) the sensitivity to detect BRCA1-type BC is 87.1% (27 out of 31) with a 51.2% specificity (22 out of 43) using a cut-off of ≥2 TDs.

### 3.3. Genes Affected by TDs in BRCA1-Type Samples

To investigate whether the transcribed genes that show TDs might have a functional role in the BRCA1-type BCs, we evaluated all 164 identified TDs (Table S1). In total, 141 genes were affected by a TD with 17 genes recurrently affected (Table 1). Next, we performed an over-representation analysis [21] to investigate common functional roles for the 141 genes. In total, 8 pathways were found to be significant (FDR < 0.05, details in Table S2), but had very broad descriptions: ‘nucleoplasm’ and ‘nucleus’ (Gene Ontology [GO] cellular component), ‘protein- and histone binding’ (GO molecular function) and ‘focal adhesion’, ‘pathways in cancer’, ‘small cell lung cancer’ and ‘prostate cancer’ (KEGG).

**Figure 4:**
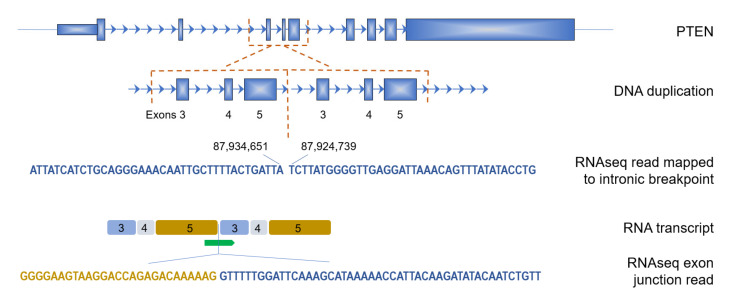
Example of a TD in *PTEN*. DNA breakpoints were identified before exon 3 and after exon 5, leading to a TD of these 3 exons. In the RNAseq data, sequence reads were identified spanning both the intron breakpoints as well as the resulting transcriptome junction of exon 5 to exon 3. Introns are indicated by the arrowed lines and are not to scale. Coordinates are from the GRCh38 reference sequence.

Next, we investigated the genes that were recurrently affected (Table 1). To check which of these recurrent genes were significantly enriched in the BRCA1-type BCs, we compared the frequencies in the BRCA1-type versus the non-BRCA1-type group using a Fisher’s exact test. After correcting for multiple testing, only *PTEN* was found significantly more often affected by a TD (FDR < 0.05) and exclusively in the BRCA1-type group. Three separate samples had a TD in *PTEN*, all of which having a different set of exons that are duplicated. In addition, all were fully concordant with the known DNA breakpoints in the WGS data of these BCs (Table 2). Additional details are shown in Figure 4 for the BC where exons 3, 4 and 5 of *PTEN* are duplicated, including a read spanning the non-canonical junction of exon 5 to 3 of mature mRNA. As our samples were processed using a ribosomal depletion method rather than a poly-A selection step, pre-mRNA was also sequenced. In the sample shown in Figure 4, intronic RNA reads were identified exactly covering the DNA breakpoint where the TD was reported. Figure S2 shows further details of this intronic breakpoint read. 

## 4. Discussion

Since a major characteristic of *BRCA1*-mutated breast cancer is an overabundance of small TDs [14], we here describe a method to identify transcripts with TDs using RNAseq and applied this method to a large cohort of BC samples. We showed that the number of TDs identified in RNAseq data strongly associated with HRD, specifically of the BRCA1-type. We additionally show that among the affected genes, *PTEN* was found significantly recurrent and exclusively in three BRCA1-type samples.

It proved possible to find sequence reads in RNAseq data that match to a configuration of tandem duplicated exons in a transcript. Although the exact mechanism on how these repeated exons are formed cannot be determined from RNAseq alone, we expect these to be mainly derived from TDs occurring on the genome. It is important to realize that only small TDs are expected to disrupt the gene structure; large duplications will yield extra copies of a complete gene, without affecting the intragenic transcript. Thus, a priori TDs identified via RNAseq are more likely to be caused by a BRCA1-type HRD than other mechanisms. Of note, in depth experiments showed how replication restart mechanisms at stalled replication forks, with a role for end joining or microhomology-mediated template switching, form the basis of generating TDs in *BRCA1* mutant cells [15]. Consistent with their different roles in HR repair, BRCA2-type cancers do not display these small TDs, but very specifically harbor small (<10 kb) deletions [14]. Another possible mechanism explaining how TDs appear in RNAseq data, independent of genomic SVs, could be some form of atypical splicing that would lead to duplications of exons in the transcript; to the best of our knowledge this has never been reported. Finally, we can’t exclude false positives; intricate errors in the complete process of the RNAseq protocol may give rise to erroneous mapping or otherwise sequencing errors. Matching our identified TDs from RNAseq with reported SVs in the genome of the respective samples, we found that indeed not all TDs found in RNAseq showed a corresponding TD event on the DNA of the tumor. Of note, the WGS sequence coverage was potentially too low to detect all possible SVs (median coverage was 40×).

Regardless of these aspects, we showed that the number of identified TDs was significantly higher in samples classified as HRD (by HRDetect) and specifically samples of BRCA1-type (by CHORD). It must be noted that although these algorithms appear very robust [8,16], there is no general agreed-upon “gold-standard” to classify HRD. However, an ROC analysis using the TD score showed a significant, high concordance with the BRCA1-type classification of CHORD. The chosen cutoff of at least 2 TD events shows a high sensitivity (88% in all samples and 87% in ER-negatives) but we recognize that this threshold needs to be validated in independent samples before diagnostic credibility is to be given to it. For example, sequencing depth of the RNA will be a major component in establishing a reliable threshold. Since it is a prerequisite to have a specific junction read in order to identify a TD, such reads are quite rare when considering all reads mapped to a gene. Thus, with a too shallow sequencing depth these rare junction reads may be missed. This in turn will influence the number of identified TDs in a sample and ultimately the threshold to establish the BRCA1-type BC from RNAseq. 

The limitation of the current method is that we are effectively measuring one of the genomic scars HRD BC samples show, albeit ‘small TDs’ is a key feature with the second highest weight in both the HRDetect and CHORD algorithms. Further approximation of the genomic features the CHORD algorithm uses (i.e., SBSs and IDs) is hindered by the absence of sequenced matched normal RNA, making the identification of somatic SBSs and IDs troublesome. So, by just scoring TDs, we do see some HRP samples having elevated numbers of TDs (both on DNA and RNA) and conversely BRCA1-type samples (via CHORD) with no (*n* = 1) or just 1 TD (*n* = 3) in RNAseq. We think these disadvantages are a reasonable trade-off since RNAseq analyses are much less costly and technically less complicated compared to full WGS of both tumor and matched normal tissue to establish all necessary genomic features. Although the current dataset of 266 BC cases is sufficient at this stage for identifying TDs in RNAseq data and relating the TD-score to CHORD, results from additional independent datasets would be required to determine clinical applicability. We have therefore tried to confirm our results in the metastatic breast cancer cohort of the Center for Personalized Cancer Treatment [16,23,24]. However, in this cohort of 160 samples for which RNAseq data was available only 9 cases were of the BRCA1-type. Although the BRCA1-type samples had a significantly higher TD-score compared to the other samples (median of 6 vs. 0; MWU *p* = 6.9 × 10^−3^), we consider the dataset too small to represent a proper validation. In summary, with the knowledge that no golden-standard HRD test exists to identify patients that may benefit from PARP inhibitors, and that about half of the HRD patients may be missed when solely focusing on bi-allelic *BRCA1* or *BRCA2* inactivation [16], we argue that standardized studies are needed to evaluate if the balance tips in favor of the precision of WGS or the ease-of-use of RNAseq to fully establish the (cost-)effectiveness of a HRD test.

Overrepresentation analysis on the genes affected by TDs identified enriched functional roles in ‘nucleoplasm’ and ‘nucleus’, ‘protein- and histone binding’, ‘focal adhesion’, ‘pathways in cancer’, ‘small cell lung cancer’ and ‘prostate cancer’. As TDs occur a priori at random, the observed enrichment in the rather general functional roles is likely a reflection of the tumorigenic selection pressure. Interestingly, we identified TDs in breast cancer driver genes *CREBBP*, *KMT2C*, *MSH2*, *PTEN*, *RB1*, *RUNX1*, and *SETD2* [14]. 

Most interestingly, we identified recurrent TDs in the *PTEN* gene, exclusively in BRCA1- type HRD samples. The TDs in the three samples consisted of different exon combinations, but all targeted the phosphatase domain of PTEN. Moreover, one of these three samples did not harbor a *BRCA1* mutation despite being designated as BRCA1-type. This may suggest a dependence for BRCA1-type HRD cancers on *PTEN* inactivation. In this respect, Saal et al. [25] showed that PTEN loss is more frequent among basal than non-basal BC, particularly among BCs from *BRCA1* mutation carriers (54.3%, 13.4% and 82.4%, respectively). In fact, loss of PTEN among BCs from *BRCA1* mutation carriers has been associated with gross or structural *PTEN* mutations rather than *PTEN* coding mutations which fits our finding. Furthermore, the authors also suggest a model in which loss of the second allele of *BRCA1* and consequently HRD leads to disruption of *PTEN*, which is then clonally selected [25,26]. Since *PTEN* inactivation has been associated with HRD and response to PARP inhibitor therapy itself [27,28,29], an alternative scenario could be that the HRD phenotype was caused directly by the disruption of *PTEN* via the TD, at least in the BRCA1-type sample that was wild-type for *BRCA1*. In this scenario the TD in *PTEN* preceded the HRD phenotype. Peng et al. take this scenario a step further by showing that *PTEN* inactivation can rescue BRCA1-driven HRD in MCF-10A cells [29]. However, the role of PTEN in homologous recombination repair and by extension, a possible role in PARP inhibitor resistance mechanisms, awaits further validation. 

## 5. Conclusions

We here developed a method to classify BRCA1-type BC with high sensitivity from RNAseq data by detecting small TDs present in transcripts. Whether or not the TDs detected via RNAseq are all caused by a TD on the genome, or via aberrant splicing or other means, these rearranged transcripts are clearly associated with BRCA1-type BC. Moreover, we show an enrichment of TDs occurring in cancer driver genes, particularly in the *PTEN* gene among BRCA1-type BCs. This suggests a role for *PTEN* inactivation in the development of BRCA1-type BC that needs to be further investigated. Importantly, RNAseq opens a new way into identifying patients that may have defective HR repair, specifically of the BRCA1-type. Such patients may benefit from PARP inhibitor anti-cancer therapy. Moreover, since BRCA1- and BRCA2-type BCs may utilize different resistance mechanisms, specifically identifying the different types of HRD defects, rather than HRD itself, may prove valuable for the clinic. In this respect, extending on our method by allowing identification of additional HRD defects via RNAseq, such as BRCA2-type HRD and the defects identified by Hussmann et al., would be worthwhile to pursue [14,30].

## Figures and Tables

**Table 1:** Genes recurrently affected by a TD in BRCA1-type BC. The FDR is calculated using the Hochberg method.

Gene	N of BRCA1-Type Samples with a TD	N of BRCA2-Type & HRP Samples with a TD	Nominal *p*-Value	FDR
*POMT1*	4	13	0.257	0.627
*ASPH*	3	12	0.431	0.627
*CDKAL1*	3	1	0.008	0.119
*PTEN*	3	0	0.002	**0.034**
*RAP1B*	3	4	0.052	0.26
*AACS*	2	0	0.017	0.119
*AKT3*	2	1	0.048	0.26
*AMY2B*	2	8	0.627	0.627
*CREBBP*	2	0	0.017	0.119
*EZH2*	2	0	0.017	0.119
*KIAA1217*	2	0	0.017	0.119
*LRP6*	2	0	0.017	0.119
*NPM1*	2	4	0.184	0.627
*PPP6R3*	2	0	0.017	0.119
*PRPSAP2*	2	0	0.017	0.119
*TTC23*	2	0	0.017	0.119
*ZNF562*	2	0	0.017	0.119

N, number; TD, tandem duplication; HRP, homologous recombination repair proficient; FDR, false discovery rate. Bold indicates the significant finding.

**Table 2:** Recurrent TDs in *PTEN*. Breakpoint coordinates are based on human reference genome GRCh38.

Sample	N TD Read-Pairs	Size (bp)	Start of Region (Exon N)	End of Region (Exon N)	Effect on Protein	DNA 5’ Breakpoint	DNA 3’ Breakpoint
p1	6	7739	3	5	out of frame	87,924,739	87,934,651
p2	13	2206	4	5	out of frame	87,926,191	87,941,211
p3	2	239	5	5	in frame	87,932,213	87,935,777

N, number; TD, tandem duplication; bp, base pairs.

## Data Availability

Genome and transcriptome data are available through the European Genome Phenome Archive (https://ega-archive.org/) under accession number EGAS00001001178.

## Supplementary Materials

The following supporting information can be downloaded at https://www.mdpi.com/article/10.3390/cancers14030753/s1. Figure S1: Distribution of TD-score; Figure S2: Annotated screenshot of Integrative Genomics Viewer (IGV) showing part of chromosome 10; Table S1: Genes affected by TDs in BRCA1-type samples; Table S2: Over-represented pathways of genes affected by TDs in BRCA1-type samples.
