# Supplementary material for: Identifying Transcripts with Tandem Duplications from RNA-Sequencing Data to Predict BRCA1-Type Primary Breast Cancer
Source: Cancers (Basel). 2022 Jan 31;14(3):753. doi: 10.3390/cancers14030753 (PMC8833645; doi:10.3390/cancers14030753)
Supplement: Supplementary file 1 [file cancers-14-00753-s001.zip › Supplementary Figures 1-2.pdf]

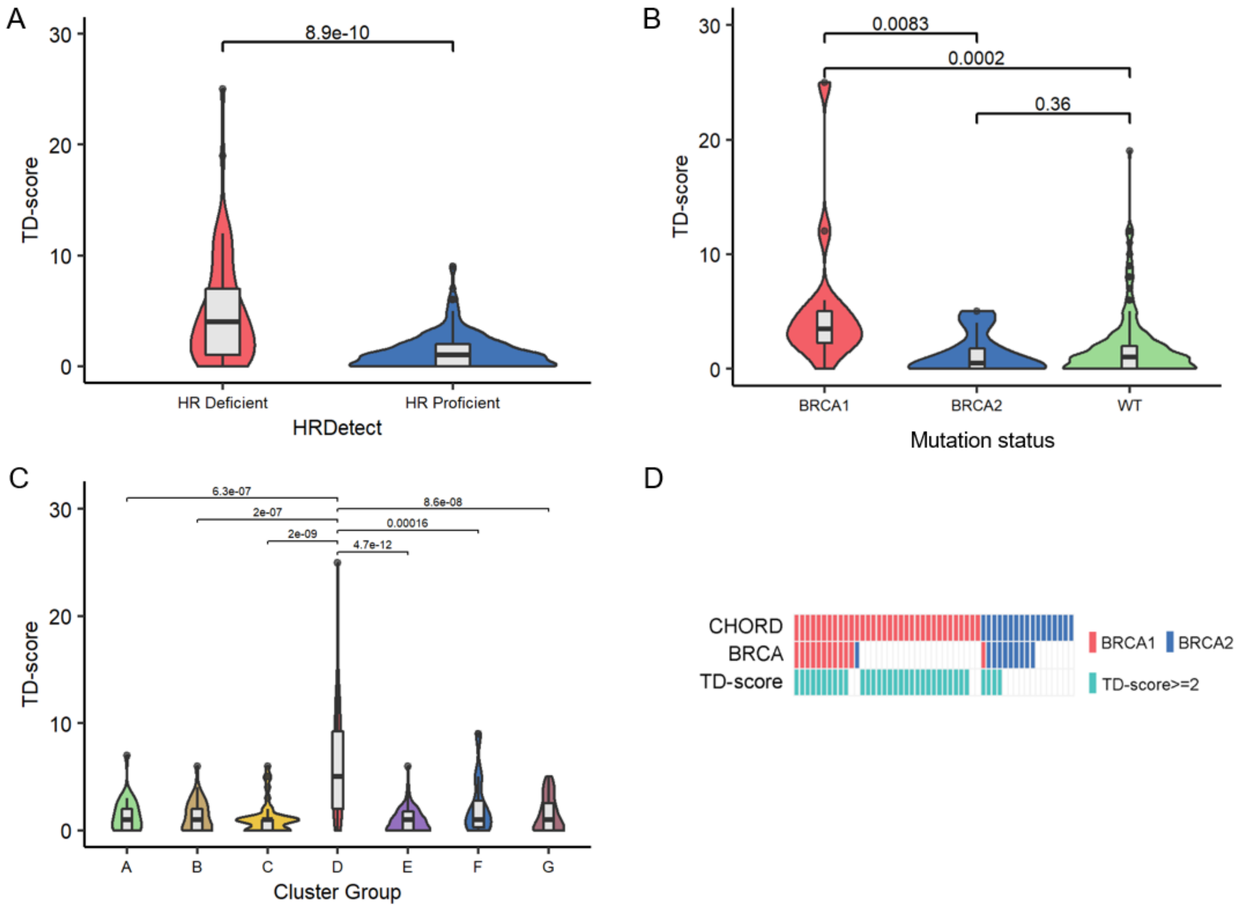

Figure S1. Distribution of TD-score according to HRDetect (A), *BRCA1* or *BRCA2* mutation status (B) or by the groups clustered using rearrangement signatures, where *BRCA1*-mutated samples are the vast majority in group D [14] (C). *P*-values are from the Mann-Whitney U test. (D) Overview of CHORD score, *BRCA1* or *BRCA2* mutation status and TD-score. CHORD BRCA1-type/BRCA2-type and *BRCA1* or *BRCA2* mutation status are in red and blue respectively, whereas TD-score ≥2 is in aqua.

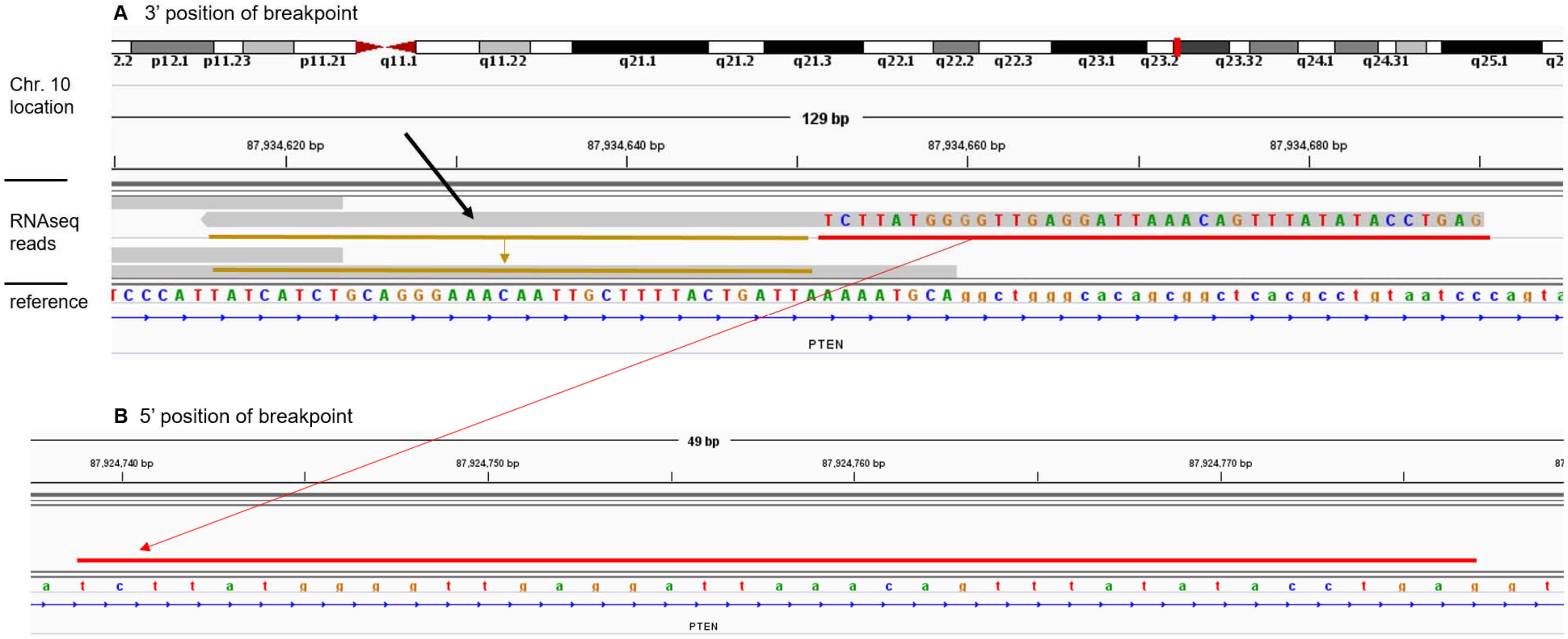

Figure S2. Annotated screenshot of Integrative Genomics Viewer (IGV) showing part of chromosome 10. The intronic sequence read (black arrow) in PTEN partly maps to the 3' DNA breakpoint (A, yellow underlined part of read) while the remainder part of the read (red underlined) does not match the reference sequence. This sequence however fully fits to the 5' position of the DNA breakpoint (B). GRCh38 human reference genome coordinates are used.
